# Erosive nappy erythema following sunitinib intake

**DOI:** 10.11604/pamj.2013.14.46.2235

**Published:** 2013-02-01

**Authors:** Saoussane Kharmoum, Hassan Errihani

**Affiliations:** 1 Department of medical oncology, National institute of oncology, Rabat Morocco

**Keywords:** Erosive, erythema, sunitinib, antiangiogenic drug

## Image in medicine

**Figure 1:**
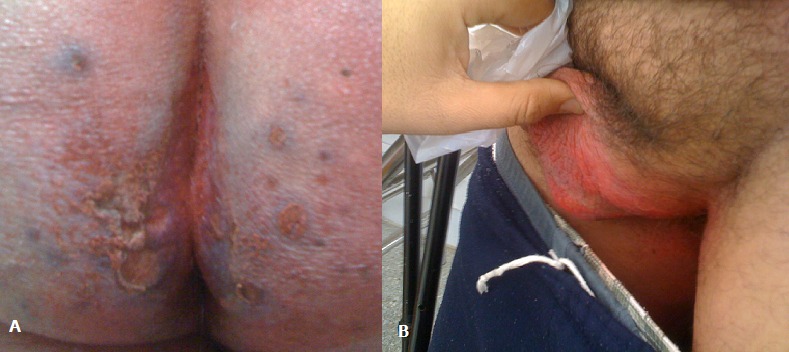
Erosive nappy erythema (A) and scrotal erythema (B)

We report on the case of a 54 years old man treating for a metastatic renal carcinoma who had experienced a dermatological toxicity referring to sunitinb intake. The antiangiogenic drug was delivered at a daily dose of 50mg for 4 weeks followed by two weeks of rest. By the second week of treatment the patient presented a nappy erythema evolving towards erosive lesions bleeding at mild friction and extending to the perianal and scrotal area, the process fulfilled the maximal intensity at 4 weeks and improved after the discontinuation of sunitinib. The patient refused the reintroduction of the drug. No skin biopsy was developed seeing that the lesions disappeared 4 weeks later. Figure 1

